# Notes and Comments

**Published:** 1923-12

**Authors:** 


### NOTES AND COMMENTS.

### THE MINISTRY AGAIN IN DANGER.

ONE of the indirect consequences of the last General Election was to leave the Ministry of Health without a head, and during the present year it has had three chief officers, all of them transient, if not embarrassed, phantoms. It is now once more in danger. Should the Government be defeated at the polls there will be a fourth Minister in twelve months, and even should it return to power the shuffling of portfolios which takes place on such occasions leaves no certainty that there may not be a new appointment to suit the exigencies of Cabinet-making. But we sincerely hope that in the latter event Sir William Joynson-Hicks (provided always that he retains his seat) will be given the chance which he has had so little opportunity of using. Much of his time during his short reign in Whitehall has been occupied by the troublesome panel question, now happily settled for the moment; but there are many others of the heterogeneous collection of subjects with which the Ministry is concerned that are pressing for settlement. No executive department of Government can ever have been so luckless as the new Ministry of Health—a Ministry which, more than most, needs a settled policy and time in which to carry it out. Prime Ministers will have to learn that a great Department which has for its mission the care of the health of the community in the widest sense of the words must not be treated as a convenience, or used to make stop-gap appointments as though it were the Privy Seal or a mere Under-Secretariat.

### BEGINNING AT THE BEGINNING.

ONE child in every three in the elementary schools of this country is suffering from a severe degree of dental disease; one in five has defective vision; four hundred thousand others are either deformed, anæmic, or insufficiently or improperly fed. This is the more than disquieting story told by Sir George Newman in his report on the Health of the School Child in 1922. Yet it is a far better story than could have been told a very few years ago, thanks to the great work which is being performed by the School Medical Service. How successful that work has been we may form some notion from the substantial proportion of children who are now leaving school with sound teeth. Unhappily by the time children reach school they have only too often already contracted some physical disability. They are born healthy in the proportion of 80 or 90 per cent., yet when they begin their "schooling," 35 to 45 per cent. of them are found to be unsound in ways which could have been prevented or cured. We shall therefore have to begin at the beginning if we are to save trouble later on, and to this end we must have closer co-operation between the Infant and Child Welfare Centre, the Day Nursery, and the Nursery School. The importance of this early care is emphasised by the unwelcome fact that the defects most marked in elementary school children are likewise the commonest in the secondary schools where, oddly enough, the physical training is "exceptionally bad." The teaching of hygiene in training colleges appears to be almost equally unsatisfactory, and it is obvious that we cannot expect the principles of health to be well taught by those whose own ignorance of them is profound.

### EFFLUVIA AND EFFLORESCENCE.

THE worse the smells, the more glorious the architecture; get rid of the smells and architecture becomes a banality. This, in a nutshell, was the argument of Major Barnes, the Vice-President of the Institute of British Architects in a Chadwick Lecture. In the fourteenth century, when the Thames and the Fleet were becoming so foul that the King issued a proclamation prohibiting the throwing of filth into their waters, Gothic architecture reached its finest efflorescence; in the nineteenth, when the battle of the public health was fought and won, architecture was at its lowest ebb. It is a piquant antithesis which might be applied to other arts than architecture. There is no necessary connexion between art and that sewer gas which has just been pronounced harmless; it is even possible for artists to be born in a sanitary State. Our ancestors probably suffered less than we do from insanitary conditions because they were an open-air people; but if we may believe one-half the stories of the chroniclers about the ravages of pestilence, they suffered badly enough. We must not be unduly alarmed at the fear that the healthier we grow the less artistic shall we become. Sanitary science has come to stay, and it is certain that art is not yet dead. Even architecture has of late years been looking up, while "boetry and bainting" have their neophytes, and occasionally their true practitioners, in greater volume than ever before. It may even be that a higher average of bodily health may bring with it such an increase of the artistic faculty that future generations may look upon Dr. Chadwick as the real begetter of a new Golden Age of Art.

### INTERNATIONAL HEALTH.

THE Annual Report of the International Health Board of the Rockefeller Foundation, of which we print a brief summary this month, brings home very forcibly the value of combination in the conquest of disease. This point was strongly emphasised in an address given before the League of Nations Union the other day by Dr. Norman White, Head of the League's Epidemic Commission. Dr. White has had a unique experience in this field of work, and during his recent visit to the Far East he was much impressed by the willingness to co-operate which everywhere exists. The East can teach as well as learn from the West. He was profoundly impressed by the excellence of Japan's public health service—his visit was, of course, before the earthquake—and considers Japan's system of the notification of disease particularly efficient and her vital statistics the most satisfactory bit of compilation in the world. Dr. White spent some time in Poland during the typhus epidemic and was instrumental in helping some of the Russian doctors once more to get into touch with medical opinion in the rest of Europe. The aim of the League is always to support and strengthen local organisation, not to replace it, and little is known to the general public of this branch of its work. It is clear, however, that it has a great opportunity before it. We understand that so far its activities have lacked adequate support, but, that forthcoming, it should do much not only in combating the various ills from which all nations alike suffer, but in cementing the ties which bind them together. Thus its health work may yet be one of the League's greatest contributions to peace.

### ONE OF THE PRIMITIVES.

"HE who lives a good life," says the ballad, "is sure to live well." Too often that is a *non sequitur*, and "living well" is assuredly not the best preparation for living long. But the late Mr. Thomas Pridgin Teale, whose death we record with regret, was a man who not only lived well, in the best sense of the phrase, but saw his ninety-second birthday. His life was one of concentrated usefulness, alike as surgeon and sanitarian. He was, indeed, one of the pioneers of practical domestic sanitation, and his book, "Dangers to Health," first published more than forty years ago, with its coloured plates and their menacing arrows showing sewer-gas rising from ill-jointed pipes and badly-trapped gullies, still holds much of its value. It is true that we are just now being assured that, instead of being regarded with disgust, sewer-gas ought to be given a certificate of character, and that most traps entrap nothing. However that may be, Pridgin Teale's insistence upon the vital importance of perfect plumbing has borne good fruit. The grate which he invented—it is still known by his name—was an early and successful attempt (Alderman Mechi was a little before him) to disestablish the high hob-grate in favour of a slow-combustion stove which warms the room and not the chimney. In these respects he was one of the primitives of sanitation. He did not invent the science, but he expanded and illustrated it, and his name will hold an honoured place in its history. He was too good a doctor and too level-headed a man to imagine that sanitary salvation is to be found in radiators. His grate is not so pretty as the old and elegant hob, but it has saved a great many cold feet and avoided millions of colds in the head.

### ASMODEUS AT LARGE.

"TO-MORROW," M. Edouard Bellin has been telling the Society of Arts, "television will be one of the realities." If he should approve himself a true prophet, he and his fellow-adepts will have inflicted upon the world a scourge even more to be dreaded than the threatened replacement of the railway by the aeroplane. "Television" is scientific jargon for the ability to see anybody at any moment, however distant, by the use of wireless. When this comes to pass no one will be more furious than the professional American humorist, so many of whose stock jokes are based upon men's assurances to wives that they are "detained at the office" when, in truth, they are enjoying life elsewhere in circumstances of greater freedom and less responsibility. When the creditor can actually see his distant debtor in his Rolls-Royce as well as speak to him, when the jealous *fiancée* can keep watch upon the divagations of the flighty young man at a distance, or perhaps even in another clime, the mistress acquaint herself with the progress of the domestic work in her absence, the whole world will live in a glass case. The demands made upon the nerves by modern life are already so serious that human nature may well quail at a prospect which threatens the last reserves of privacy. Perhaps, after all, M. Bellin may be a false prophet, or merely be taking a malicious pleasure in making our flesh creep. But the curious thing is that these terrible scientific inventors really seem to think that they are conferring benefits upon humanity by making life distinctly less worth living.

### POPULAR HEALTH LITERATURE.

AT a meeting of the National Baby Week Council, Dr. O. K. Wright has been criticising popular health literature, and exposing some of the ineptitudes which too often mar it. He instanced a poster which intended to condemn the "baby's comforter," but actually "made out a good case for its retention," while "an unskilful advertisement for vaccination showed a baby suffering from a mild attack of small-pox." That posters of this kind give "a great deal of unattractive advice," and are "calculated to arouse the antagonism of the reader," is, unhappily, true enough. We seem to need a censorship of this type of literature, though it might not be easy to fix upon the censor, while it would be exceedingly difficult to make well-meaning enthusiasts amenable to him. The societies and organisations which seek to interest the public in health matters could, however, do much to prevent this mistaken propaganda. The British public is critical in such matters, and can display a nice taste in sarcasm, and it is always bad propaganda to afford the enemy an opportunity to blaspheme.

### THE HEALING SUN.

FROM very early ages mankind has known something of the healing power of the sun, but we have been long in learning to use it as a regular curative agent. Sir Henry Gauvain's lecture at the Society of Arts on "The Sun Treatment" as practised at the Alton Cripples' Home gave some remarkable indications of what he himself described as the "almost uncanny" things it can do. That children "in a terrible condition" of tuberculosis and deformity can be "practically cured" in six months' time by the sun treatment is, indeed, "one of the most romantic chapters in medical science." Sir Henry is satisfied that we are only at the beginning of our knowledge of what the sun can do in the curing of disease, and the results of his further experience and experiments will be looked for with as much eagerness by the general public as by men of science. One reason why a summer holiday does us good is that we are much exposed to the sun as well as to the air, and if we could regulate its shining as we regulate the electric light we should be in command of what is, perhaps, the best of all medicines. Eternal fierce sunshine is not agreeable, as residents in hot countries know only too well, nor would plentiful daily sunshine make us immortal; but the sun can prevent as well as cure disease, and it is to prevention that in the future we must look not merely for the prolongation of life but for making life sweet, wholesome, and useful to others as well as to ourselves.

### FEEDING WEAKLY SCHOOL CHILDREN.

THE wholesale feeding of school children at the expense of the community is an undertaking about which much may be said for and against. The feeding of puny, under-nourished children who lag behind their companions both at work and play for want of general vitality is another matter. An experiment has recently been carried out in Christiania with children of this class. They were picked out by their teachers from the various school grades and put in a separate class. Their first meal was given at school, an appetite for porridge, milk, cocoa and bread and butter having been acquired on the way thither. They were made to eat slowly and not to "wolf" their food. A substantial midday meal was also given at school, and they were told to rest for an hour after returning home. Every fortnight a class was held for the mothers, and when, three times running, a mother failed to attend, her child was automatically discharged from the special feeding class. In this way gentle pressure was put on the mothers to learn how to feed and care for their sickly offspring. So far the results have been most encouraging. Of twenty-four children selected for this experiment, twenty-two gained uniformly in weight, and many had approached the standard of height and weight qualifying them for a return to ordinary school life. As has lately been pointed out by the School Medical Officer in Christiania, Dr. C. Schiötz, the class of child provided for under this scheme is the class from which the consumptives of adolescence and adult life are recruited. If this source of recruiting can be eliminated by a little care and extra feeding in school life, many a potential consumptive may remain well for a normal lifetime.

### UNFIT FOR BABIES.

THE Minister of Health has recently notified Local Authorities of Regulations, to come into force on May 1st, 1924, prescribing the labelling and composition of dried milk. These regulations provide for the taking of samples by the Medical Officer of Health, and for the subsequent tests with a view to seeing that the composition of the milk and, in particular, the percentage of milk fat, comes up to the standards laid down. Milk affected by these regulations will require to be plainly labelled "Dried full cream milk," "Dried partly-skimmed milk" (with the addition of the words "should not be used for babies except under medical advice"), or "Dried skimmed milk," as the case may require. In the last case, the words "Unfit for babies", are to appear on the label in large type, without any comment or explanation. This is a rigid and drastic requirement, but we have no hesitation in expressing, on behalf of the 1924 babies, the view that it is a very sound requirement and their thanks to the Minister for so very proper a measur of protection.

### EFFECT OF SMOKING.

IN view of the serious import usually attached to a persistently high blood pressure, and the fact that in this, as in so many other conditions, smoking is often prohibited, some experiments recently recorded in the *Journal of the American Medical Association* are of particular interest. Bates has made complete records of the blood pressure in students who, seated comfortably meanwhile, smoked extensively while under observation. There seems to be no doubt that the smoking of a cigar or three cigarettes will to some extent raise both the blood pressure and the heart rate. On the other hand, the rise in pressure is relatively slight and, as the author points out, there are many other everyday influences which produce variations in pressure quite as great, if not greater. At first sight this might be taken to imply that, smoking being but one pressure-raising influence among many, its prohibition is hardly worth while. Nevertheless, the abuse of tobacco is something which can easily be deleted from our daily habits, and that without interference with efficiency, whereas other influences, such as active movement or performance of any one of a thousand possible energy absorbing tasks, may, if disallowed, prove a serious handicap in this competitive age.

### BETTING AND BALLOTS.

THE proposed tax upon betting, it would seem, has already been decently interred, and now the London County Council is giving attention to ballots, hospital and otherwise. To the purist the ballot may be as unlawful as betting, but at least it is legal, which is more than can always be said of betting. The Council is concerned as to the proportion of the proceeds which is taken by the promoters. Sometimes this proportion has soared as high as one-half, but it appears usually to be about 25 per cent. The cost of printing, postage and advertising is always large, and where, in addition to this, the promoters guarantee the prizes, one-quarter of the proceeds is probably not too high a return. So far as they are concerned a ballot is really a gamble, in which large sums may be won or lost. The point of view of the hospital that benefits may well be different, since it runs no risk. Wherever matters of this kind touch hospital work, it is well to remind ourselves what the true position is. All pleasures divide themselves into the good, the bad, and the indifferent; but even the good, such is human frailty, may become evil by indulgence to excess. The difference of ethical value is a difference not of kind, but of degree, and therefore the precise degree decides whether the action is good or evil. Gambling is betting carried to excess; but because only excess is evil, it argues that moderation is not.

To forbid it absolutely creates more harm than good—the lesson of Prohibition in America. The law comes in to guard against excess only by such simple regulations as is all it can hope, or should try, to enforce. Hospital ballots and the like, such as are just now in fashion, are therefore perfectly permissible, provided that the scale on which they are run is accompanied by the highest credentials in their organisers.

### THE DEVELOPMENT OF WARTS.

IF the man in the street, a school teacher, or even that superb depository of all knowledge, the senior medical student, were to be asked how warts develop, probably none of them could give an answer that would "satisfy the examiners." Recent investigations at a hospital in Vienna have thrown interesting light on the matter. It has been found that if a warty growth in the throat is removed and inoculated into scratches in the skin, warts will develop in these scratches after a certain incubation period. These warts can be transmitted from one person to another, and their rate of growth increases by such passage, and the incubation period can be shortened. These observations leave little doubt as to the microbic origin of some, if not of all, warts. Further investigations have shown that the responsible germ is very minute, passing through filters which retain germs large enough to be visible under the microscope. The lesson of these investigations is that when warts develop on the grubby paws of our offspring, cleanliness and antiseptics are indicated rather than the resigned assumption that the youngsters will outgrow these excrescences just as they outgrow their sailor suits.

### "COLOUR-HEARING" AND HEREDITY.

THE term "colour-hearing"—*l'audition colorée*—has been applied to that sense which enables some persons to associate certain sounds with certain colours. There may be other, similar combinations such as the sense of smell, or of taste with that of colours. In the past the possessors of these double sensations, synæsthesias or secondary sensations, have often been discreetly silent about them, fearing that unsympathetic friends might regard them as signs of insanity, or at least of eccentricity. Now, however, it is generally admitted that "colour-hearing" is not a morbid, but a perfectly physiological, though rare, phenomenon, and recent investigations by a Scandinavian doctor who has sought for the presence of this combined sensation in three generations of the same family, show that it is a property handed down from one generation to another, according to Mendel's laws. He found it in ten members of this family, and in the six members of the second generation who showed this characteristic the sexes were equally represented. It is a curious fact that a person blessed with this gift correlates the same sounds with the same colours throughout his life—a "blue sound" in boyhood does not give place to a "red sound" in old age. He is sometimes in the curious position of deciding whether a chord is discordant or not by the colour-sensation to which it gives rise.

### RESTORING FADED RECORDS.

THOSE associated with the older hospitals and charities inevitably meet with old and interesting written records, and the wish is often expressed that these things could be easily and safely restored. Our attention is held, therefore, by a note in the *Pharmaceutical Journal* on the simpler methods of restoring faded writing. In summary the suggestions are as follows. Although if the original ink was a coal-tar colour, frequently it cannot possibly be restored, yet writings made with methyl-violet can often be brought out by simple exposure to ammonia gas. Faded inks which contained iron can be rendered legible by treatment with tannin, ammonium sulphide, potass. ferro-cyanide, ammonium sulphocyanide or sodium salicylate. The routine outlined is that the paper should first be placed between clean sheets of writing paper and pressed with a heated iron. This alone may bring out the writing; if it fails, expose the paper to sunlight and, if this also is unsuccessful, apply, as a trial, a solution of one of the substances named to one corner of the paper and iron as before. The whole can be treated later. These plans do not, of course, exhaust all the possibilities in restoration of faded writings, but their simplicity may make them valuable to those who care for and treasure records of the past.

### MICROSCOPING THE LIVING EYE.

IT has long been possible to examine under the microscope sections made from tissues removed at operation—dead tissues, in short. But the application of the high-power microscope to the living organs is something relatively new. There is always the possibility that the condition of tissues may not, after their removal from the body, be exactly the same as they were in life and, although the new method of examination is available at present for one organ only—the eye—great possibilities are opened up. A microscope magnifying more than one hundredfold can be focussed upon the various layers of the living eye, when used in conjunction with a new invention known as a slit-lamp. This lamp carries a small arc light inside a tube, at the end of which is a tiny slit only half-a-millimetre wide. The image of the luminous arc is thrown through the slip and viâ certain lenses into the eye. The lamp therefore emits a minutely narrow streak of light which penetrates to the back of the eye with practically no discomfort to the patient and none of the glare associated with the older methods. When the microscope is focussed upon the parts illuminated by this streak of light, the areal ighted up appears to be of considerable breadth and individual cells can readily be seen and studied. Practical use of the lamp is fully described by Dr. Mulder in a recent number of the *South African Medical Record*, and it is obvious that the possibilities are very wide. As he points out, diagnosis can be made earlier, minor defects are recognised which could not be seen before, and by this means it is possible to judge much better whether and in how far an eye has recovered from injury or disease.

